# Tetanus as Cause of Mass Die-off of Captive Japanese Macaques, Japan, 2008

**DOI:** 10.3201/eid1810.120503

**Published:** 2012-10

**Authors:** Tomomi Nakano, Shin-ichi Nakamura, Akihiko Yamamoto, Motohide Takahashi, Yumi Une

**Affiliations:** Azabu University, Kanagawa, Japan (T. Nakano, S. Nakamura, Y. Une);; and National Institute for Infectious Diseases, Tokyo, Japan (A. Yamamoto, M. Takahashi)

**Keywords:** Clostridium tetani, bacteria, Japanese macaque, Macaca fuscata, outbreak, tetanus, zoonoses, Japan

## Abstract

In 2008 in Japan, 15/60 captive Japanese macaques died. *Clostridium tetani* was isolated from 1 monkey, and 11 had tetanus-specific symptoms. We conclude the outbreak resulted from severe environmental *C. tetani* contamination. Similar outbreaks could be prevented by vaccinating all monkeys, disinfecting housing areas/play equipment, replacing highly *C. tetani*–contaminated soil, and conducting epidemiologic surveys.

Tetanus is a wound infection caused by a potent neurotoxin produced by *Clostridium tetani*. The bacterium is difficult to isolate, and no pathologically characteristic lesion is present during infection; thus, tetanus diagnosis is based on tetanus-specific clinical symptoms (*1*–*4*). Tetanus is a highly lethal zoonosis, and cases usually occur sporadically. Outbreaks among humans have occurred only after earthquakes and tsunamis (*4*). We report on an outbreak of tetanus in 2008 among a captive colony of Japanese macaques (*Macaca fuscata*) in Japan.

## The Study

**Figure 1:**
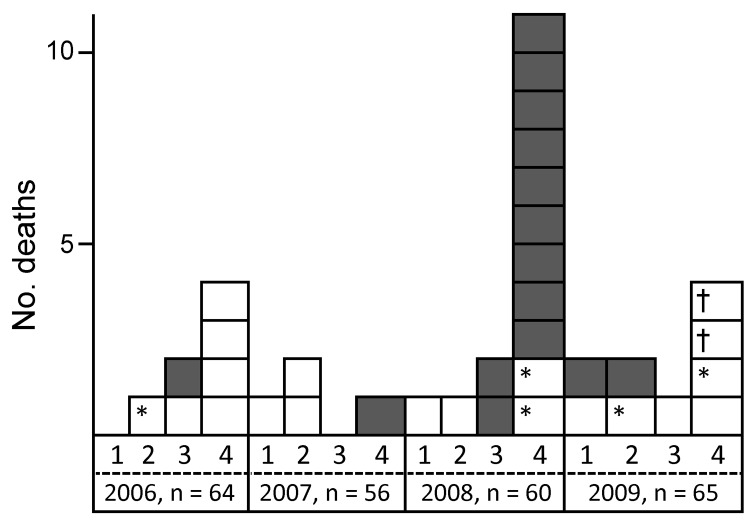
Number of deaths during 2006–2009 among macaques (*Macaca fuscata*) housed in an animal facility in the Kantou area of Japan. Grey boxes, monkeys with tetanus-specific clinical symptoms; white boxes, monkeys without tetanus-specific clinical symptoms. 1, January–March; 2, April–June; 3, July–September; 4, October–December; n, total number of monkeys. *Juvenile animal; †Accident at time monkeys captured for vaccination (death due to hyperthermia).

In 2008, deaths suddenly increased among Japanese macaques housed in a facility in the Kantou area of Japan. At that time, the facility, which had been in service for >40 years, housed ≈60 macaques, 15 (25%) of which died. This mortality rate was much higher than that during 2006 (10.9%, 7/64 monkeys), 2007 (7.1%, 4/56), 2009 (13.8%, 9/65), 2010 (5.2%, 3/58), and 2011 (5.7%, 4/70) (Figure 1). A total of 42 monkeys died during 2006–2011, and investigations at the time of death showed that 14 of the monkeys had tetanus-specific symptoms: 1 of 4 that died in 2007, 11 of 15 that died in 2008, and 2 of 9 that died in 2009). Nine of the 11 monkeys that died with characteristic symptoms of tetanus in 2008 died during the breeding season (November and December). Thus, the observed number of presumed tetanus cases during the 2008 breeding season (9/60) was 8.4× greater than the number during the 2007 breeding season (1/56).

The soil in the monkeys’ enclosure was clay-like and without vegetation. In 2008 before the increase in deaths, there were no changes in maintenance procedures, such as feeding, at the facility and no evident pathogenic contamination of the monkeys’ food or environment.

**Figure 2:**
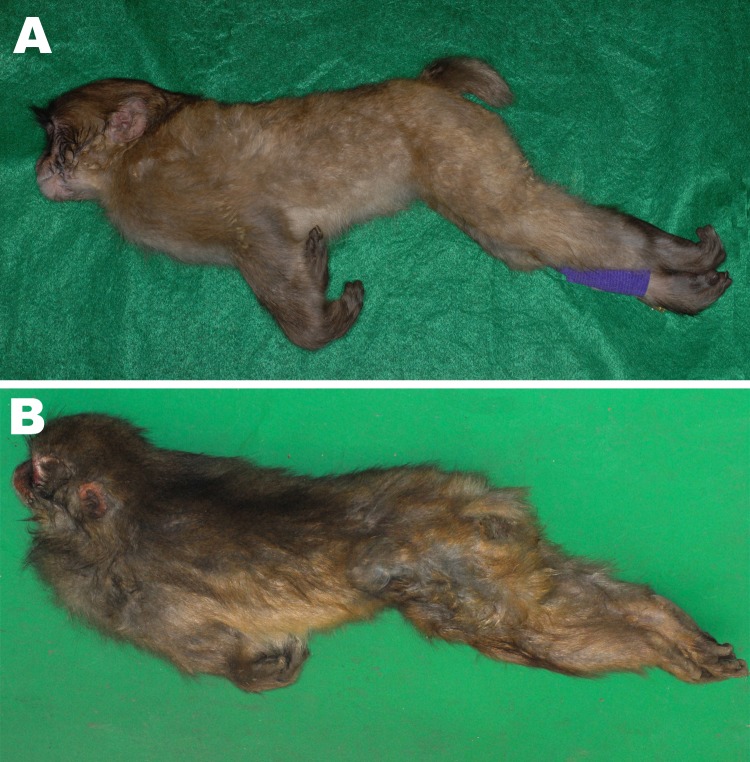
A) Opisthotonos as a tetanus-specific clinical symptom in a 1-year-old male Japanese macaque (*Macaca fuscata*). B) Opisthotonos with severe rigid posture in an adult male Japanese macaque.

We performed necropsies on 3 monkeys (animal nos. 1, 2, and 3) that died 5, 2, and 3 days, respectively, after the onset of symptoms. At death, all showed a specific posture: the jaw was elevated, the back straightened, and the tail tightly stretched; the forelimbs were crossed in front of the body with the wrists bent; and the hind limbs were extended backward (Figure 2). Rigidity was abnormally severe and did not remit after death; at necropsy, the mouth was difficult to open. Congestion of the visceral organs and pulmonary edema were noted, but there were no findings to suggest poisoning, such as foreign bodies in the stomach or erosive changes in the gastrointestinal tract. No wound that might have led to infection was found in monkeys 1 or 2, but a lesion with purulent incrustation was present on a toe tip on the right hind limb of monkey 3. *C. tetani* was isolated from this lesion, and the tetanus toxin gene was detected by PCR. A mouse toxicity test confirmed tetanus toxin activity.

We obtained samples from the soil in monkey enclosures, from wooden playground equipment, and from the soil surrounding the enclosures and tested them for *C. tetani*; 67%, 75%, and 53% of the samples, respectively, were positive for *C. tetani*, indicating marked contamination. *C. tetani* was not isolated from the monkeys’ food or from soil sampled >1 km from the facility. We performed pulsed-field gel electrophoresis on isolates from the soil at the facility and from monkey number 3, and the results were identical, showing >90% homology.

On 3 occasions (October 27 and December 17, 2009, and December 8, 2010), macaques housed in the facility (total 65) were intramuscularly administered 0.5 mL of tetanus toxoid (Nisseiken Co., Ltd., Tokyo, Japan). In 1 monkey, the prevaccination serum level of tetanus toxoid antibody was higher than the level for tetanus prevention (0.1 IU/mL). At 51 days after the first vaccination, 83.3% (51/61) of the animals were antibody-positive, and 1 year after the second vaccination, 100% were antibody-positive. Since then, no tetanus symptoms have occurred in any of the monkeys. Caretakers for monkeys at the facility were examined at a community medical office and inoculated with tetanus toxoid.

## Conclusions

On the basis of these findings, we diagnosed the disease as tetanus, and we concluded that it was an unprecedented, large-scale outbreak. Many animal exhibition facilities in Japan maintain Japanese macaques, and tetanus has been reported in captive macaques in other countries (*5*–*7*). Results of a 5-year study (July 1, 1976–June 30, 1981) among the free-ranging rhesus monkey (*Macaca mulatta*) colony on the island of Cayo Santiago, Puerto Rico, showed a high incidence of tetanus among the monkeys during the breeding seasons, but the report did not clarify the cause (*6*).

In facilities maintaining animals, the soil is often contaminated with *C. tetani* at a relatively high rate (*1*,*2*). In the facility in Japan, *C. tetani* was isolated at a high rate from soil and from play structures. The genotype of these isolates was consistent with that for an isolate obtained from a monkey housed at the facility, suggesting that the soil was the source of the infection.

The facility has >40 years’ experience raising monkeys, and the cause of the sudden outbreak in 2008 is unclear. The outbreak was concentrated during the breeding season, suggesting that injuries sustained through fighting during the mating season in an environment with severe *C. tetani* contamination may have led to the outbreak. *C. tetani* is present in the intestinal contents of various animal species (*1*,*3*). Thus, bacteria in the feces of infected monkeys may have added to the level of indigenous *C. tetani* contamination in the soil.

In Japan, tetanus is still reported in >100 persons each year: 115 cases were reported in 2005, 117 in 2006, 89 in 2007, 124 in 2008, 113 in 2009,and 106 in 2010) (*8*). It is a highly lethal zoonosis and a disease of concern with regard to public and animal health. After tetanus was diagnosed in the monkeys, we immediately administered tetanus vaccine to monkey caretakers at the facility and thoroughly enforced hygiene practices. To prevent tetanus infection in animals and animal caretakers in such facilities and in visitors, we recommend that newborn monkeys be vaccinated, housing areas and play equipment be disinfected, soil highly contaminated with *C. tetani* be replaced, and epidemiologic surveys be conducted.

## References

[1] Becker DG, Lineaweaver WC, Edlich R, Hill LG, Mahler CA, Cox MJ, Management and prevention of tetanus. J Long Term Eff Med Implants. 2003;13:139–54. 10.1615/JLongTermEffMedImplants.v13.i3.20 14516181

[2] Brook I. Current concepts in the management of *Clostridium tetani* infection. Expert Rev Anti Infect Ther. 2008;6:327–36. 10.1586/14787210.6.3.327 18588497

[3] Novak RT, Thomas CG. Tetanus. In: CDC health information for international travel 2012: the yellow book [cited 2012 Apr 27]. http://wwwnc.cdc.gov/travel/yellowbook/2012/chapter-3-infectious-diseases-related-to-travel/tetanus.htm

[4] World Health Organization. Current recommendations for treatment of tetanus during humanitarian emergencies [cited 2012 Jan 15]. http://whqlibdoc.who.int/hq/2010/WHO_HSE_GAR_DCE_2010.2_eng.pdf

[5] Digiacomo RF, Missakian EA. Tetanus in a free-ranging colony of *Macaca mulatta*: a clinical and epizootiologic study. Lab Anim Sci. 1972;22:378–83. 4338701

[6] Rawlins GR, Kessler MJ. A five-year study of tetanus in the Cayo Santiago rhesus monkey colony: behavioral description and epizootiology. Am J Primatol. 1982;3:23–39. 10.1002/ajp.1350030103 31991997

[7] Springer DA, Phillippi-Falkenstein K, Smith G. Retrospective analysis of wound characteristics and tetanus development in captive macaques. J Zoo Wildl Med. 2009;40:95–102. 10.1638/2008-0055.1 19368246 PMC3409561

[8] Yamamoto A, Takahashi M. Clostridium tetani [in Japanese]. Nihon Rinsho. 2010;68(Suppl 6):220–3. 20942042

